# Supplementary material for: HERMES: Holographic Equivariant neuRal network model for Mutational Effect and Stability prediction
Source: ArXiv. 2024 Jul 9:arXiv:2407.06703v1. Preprint. [Version 1] (PMC11261993)
Supplement: Supplement 1 [file NIHPP2407.06703v1-supplement-1.pdf]

Appendix A

		ProTherm				*Protein G*	*VAMP-seq*			
		1BVC	*1LZ1*	*2CI2*	*2RN2*	*1PGA*	*5BON*	*2H11*	*1D5R*	*Average*
Zero-Shot	*ESM-1v*	0.49	0.49	0.21	0.27	0.24	0.48	**0.54**	0.44	0.40
	ProteinMPNN 0.02	0.50	0.56	0.43	0.19	0.48	0.47	0.40	0.52	0.44
	ProteinMPNN 0.20	0.57	0.64	0.51	0.30	0.44	0.48	0.41	0.51	0.48
	HERMES BP 0.00	0.29	0.40	0.47	0.36	0.44	0.42	0.33	0.42	0.39
	HERMES BP 0.50	0.52	0.42	0.52	0.58	0.57	0.50	0.41	0.49	0.50
	HERMES PR 0.00	0.50	0.50	0.44	0.42	0.50	0.48	0.39	0.47	0.46
	HERMES PR 0.50	0.63	0.51	0.58	0.65	0.59	0.54	0.45	0.52	0.56
Fine-Tuned	*RaSP + FT*	**0.71**	0.57	**0.65**	0.79	0.72	0.50	0.48	0.52	0.62
	HERMES BP 0.00 + FT	0.64	0.60	0.55	0.70	0.74	0.60	0.52	0.57	0.62
	HERMES BP 0.50 + FT	**0.71**	0.57	0.63	0.78	**0.76**	**0.61**	0.52	0.56	**0.64**
	HERMES PR 0.00 + FT	0.67	**0.62**	0.54	0.73	0.73	**0.61**	0.52	0.57	0.62
	HERMES PR 0.50 + FT	**0.71**	0.58	0.56	**0.80**	0.74	0.60	0.52	**0.58**	**0.64**

TABLE S1. **Pearson Correlation as prediction performance on experimental $\Delta\Delta G^{\text{stability}}$ for eight test proteins.** Same data as Figure 3.

		S669		*Ssym dir.*		*Ssym rev.*
		Per Structure	*Overall*	*Per Structure*	*Overall*	*Overall*
Zero-Shot	*ProteinMPNN 0.02*	0.42 ± 0.08	0.30	0.30 ± 0.10	0.52	0.30
	ProteinMPNN 0.20	0.47 ± 0.07	0.34	0.40 ± 0.09	0.56	0.39
	HERMES BP 0.00	0.31 ± 0.08	0.15	0.31 ± 0.09	0.47	0.21
	HERMES BP 0.50	0.40 ± 0.07	0.29	0.45 ± 0.08	0.56	0.30
	HERMES PR 0.00	0.37 ± 0.06	0.21	0.38 ± 0.09	0.53	0.17
	HERMES PR 0.50	0.44 ± 0.06	0.31	0.53 ± 0.06	0.61	0.30
Fine-Tuned	*RaSP + FT*	-	**0.39**	-	0.58	0.18
	HERMES BP 0.00 + FT	0.44 ± 0.07	0.32	0.48 ± 0.06	0.59	0.37
	HERMES BP 0.50 + FT	0.47 ± 0.07	0.38	0.54 ± 0.05	0.62	0.44
	HERMES PR 0.00 + FT	0.47 ± 0.06	0.34	0.53 ± 0.05	0.62	0.36
	HERMES PR 0.50 + FT	**0.50** ± 0.07	**0.39**	**0.59** ± 0.04	**0.64**	**0.46**

TABLE S2. **Pearson correlation with stability $\Delta\Delta G$ from S669 and Ssym datasets.** "Per Structure" correlations are computed by averaging correlations across proteins with at least 10 mutations; "Overall" correlations are instead computed across all mutations among all proteins. For RaSP we currently report only values that were reported in their paper. Ssym reverse has no Per Structure correlation since every mutant has its own structure. Standard error is shown for per-structure correlations.

		All Mutations		Single-Point Mutations	
		Per Structure	*Overall*	*Per Structure*	*Overall*
Zero-Shot	*ESM-1v*	0.01	0.19	-	-
	ProteinMPNN 0.02	0.25 ± 0.03	0.29	0.28 ± 0.03	0.33
	ProteinMPNN 0.20	0.24 ± 0.03	0.27	0.30 ± 0.03	0.33
	HERMES BP 0.00	0.21 ± 0.03	0.20	0.23 ± 0.03	0.24
	HERMES BP 0.50	0.24 ± 0.02	0.21	0.27 ± 0.03	0.24
	HERMES PR 0.00	0.26 ± 0.03	0.24	0.29 ± 0.03	0.28
	HERMES PR 0.50	0.29 ± 0.03	0.25	0.31 ± 0.03	0.29
Fine-Tuned	*HERMES BP 0.00 + FT*	0.31 ± 0.02	0.33	0.34 ± 0.03	0.39
	HERMES BP 0.50 + FT	0.30 ± 0.02	0.30	0.32 ± 0.03	0.35
	HERMES PR 0.00 + FT	**0.32** ± 0.02	**0.34**	**0.36** ± 0.03	**0.40**
	HERMES PR 0.50 + FT	0.31 ± 0.02	0.31	0.34 ± 0.03	0.36

TABLE S3. **Pearson correlation of models with Binding $\Delta\Delta G$ from the SKEMPI 2.0 dataset.** Standard error is shown for per-structure correlations. We do not show it for ESM-1v as we show previously-reported scores.

		All Mutations		Single-Point Mutations	
		Per Structure	*Overall*	*Per Structure*	*Overall*
Zero-Shot	*ProteinMPNN 0.02*	0.31 ± 0.11	0.08	**0.37** ± 0.11	**0.24**
	ProteinMPNN 0.20	0.17 ± 0.14	0.07	0.26 ± 0.14	0.19
	HERMES BP 0.00	0.15 ± 0.04	0.14	0.16 ± 0.04	0.11
	HERMES BP 0.50	0.16 ± 0.04	**0.21**	0.16 ± 0.05	0.15
	HERMES PR 0.00	0.06 ± 0.08	0.10	0.10 ± 0.07	0.08
	HERMES PR 0.50	0.07 ± 0.07	0.20	0.09 ± 0.06	0.12
Fine-Tuned	*HERMES BP 0.00 + FT*	**0.32** ± 0.07	0.14	**0.37** ± 0.06	0.21
	HERMES BP 0.50 + FT	0.27 ± 0.06	0.18	0.30 ± 0.07	0.21
	HERMES PR 0.00 + FT	0.27 ± 0.08	0.14	0.33 ± 0.07	0.17
	HERMES PR 0.50 + FT	0.21 ± 0.06	0.18	0.25 ± 0.06	0.16

TABLE S4. **Pearson correlation of models with Binding $\Delta\Delta G$ from the ATLAS dataset, using only wildtype structures.** Standard error is shown for per-structure correlations.

		All Mutations		Single-Point Mutations	
		Per Structure	*Overall*	*Per Structure*	*Overall*
Zero-Shot	*ProteinMPNN 0.02*	**0.33** ± 0.10	0.13	0.37 ± 0.11	0.23
	ProteinMPNN 0.20	0.18 ± 0.13	0.10	0.26 ± 0.14	0.17
	HERMES BP 0.00	0.29 ± 0.07	0.06	0.35 ± 0.05	0.16
	HERMES BP 0.50	0.28 ± 0.07	0.12	0.34 ± 0.08	0.17
	HERMES PR 0.00	0.29 ± 0.07	0.09	0.36 ± 0.06	0.17
	HERMES PR 0.50	0.28 ± 0.07	0.13	0.34 ± 0.08	0.16
Fine-Tuned	*HERMES BP 0.00 + FT*	0.32 ± 0.09	0.07	**0.42** ± 0.08	0.22
	HERMES BP 0.50 + FT	0.31 ± 0.10	**0.15**	0.40 ± 0.11	**0.25**
	HERMES PR 0.00 + FT	0.30 ± 0.10	0.10	0.40 ± 0.09	0.21
	HERMES PR 0.50 + FT	0.29 ± 0.08	0.14	0.37 ± 0.09	0.20

TABLE S5. **Pearson correlation of models with Binding $\Delta\Delta G$ from the ATLAS dataset, using both wildtype and mutant structures.** Error bars show the standard error.

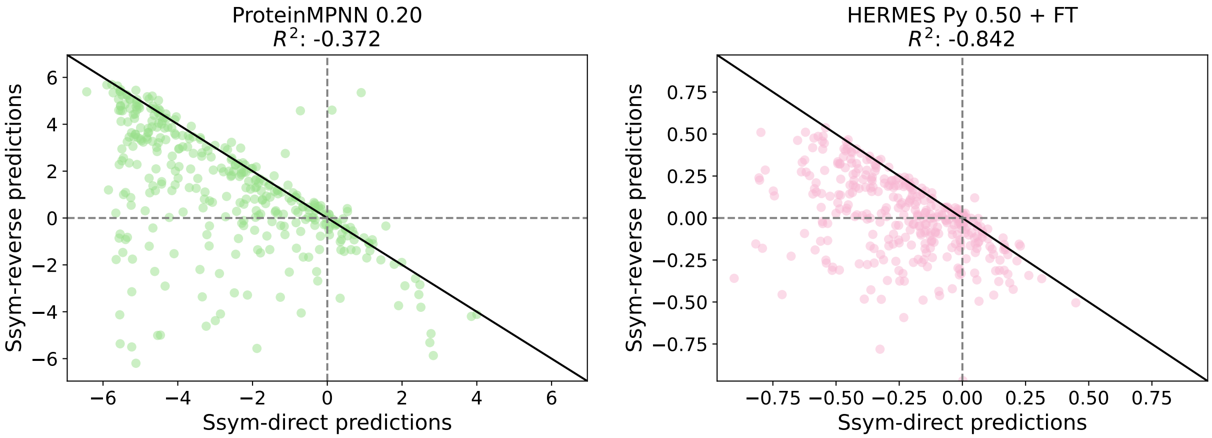

FIG. S1. **Predictions on Ssym-direct vs. Ssym-reverse.** Each point is a mutation. Prediction values are made according to Equation 2. Remarkably, predictions on Ssym-reverse are consistently under-shooting.
